# Disrupted Thalamus White Matter Anatomy and Posterior Default Mode Network Effective Connectivity in Amnestic Mild Cognitive Impairment

**DOI:** 10.3389/fnagi.2017.00370

**Published:** 2017-11-08

**Authors:** Thomas Alderson, Elizabeth Kehoe, Liam Maguire, Dervla Farrell, Brian Lawlor, Rose A. Kenny, Declan Lyons, Arun L. W. Bokde, Damien Coyle

**Affiliations:** ^1^Intelligent Systems Research Centre, University Ulster, Derry, United Kingdom; ^2^Trinity College Institute of Neuroscience and Cognitive Systems Group, Discipline of Psychiatry, School of Medicine, Trinity College Dublin, Dublin, Ireland; ^3^Mercer’s Institute for Research on Ageing, St. James’s Hospital, Trinity College Institute of Neuroscience, Trinity College Dublin, Dublin, Ireland; ^4^St. Patrick’s Hospital, Dublin, Ireland

**Keywords:** diffusion MRI, tractography, effective connectivity, Alzheimer’s disease, mild cognitive impairment, default mode network, thalamus, resting state

## Abstract

Alzheimer’s disease (AD) and its prodromal state amnestic mild cognitive impairment (aMCI) are characterized by widespread abnormalities in inter-areal white matter fiber pathways and parallel disruption of default mode network (DMN) resting state functional and effective connectivity. In healthy subjects, DMN and task positive network interaction are modulated by the thalamus suggesting that abnormal task-based DMN deactivation in aMCI may be a consequence of impaired thalamo-cortical white matter circuitry. Thus, this article uses a multimodal approach to assess white matter integrity between thalamus and DMN components and associated effective connectivity in healthy controls (HCs) relative to aMCI patients. Twenty-six HC and 20 older adults with aMCI underwent structural, functional and diffusion MRI scanning using the high angular resolution diffusion-weighted acquisition protocol. The DMN of each subject was identified using independent component analysis (ICA) and resting state effective connectivity was calculated between thalamus and DMN nodes. White matter integrity changes between thalamus and DMN were investigated with constrained spherical deconvolution (CSD) tractography. Significant structural deficits in thalamic white matter projection fibers to posterior DMN components posterior cingulate cortex (PCC) and lateral inferior parietal lobe (IPL) were identified together with significantly reduced effective connectivity from left thalamus to left IPL. Crucially, impaired thalamo-cortical white matter circuitry correlated with memory performance. Disrupted thalamo-cortical structure was accompanied by significant reductions in IPL and PCC cortico-cortical effective connectivity. No structural deficits were found between DMN nodes. Abnormal posterior DMN activity may be driven by changes in thalamic white matter connectivity; a view supported by the close anatomical and functional association of thalamic nuclei effected by AD pathology and the posterior DMN nodes. We conclude that dysfunctional posterior DMN activity in aMCI is consistent with disrupted cortico-thalamo-cortical processing and thalamic-based dissemination of hippocampal disease agents to cortical hubs.

## Introduction

Alzheimer’s disease (AD) is a chronic neurodegenerative disorder affecting approximately 6% of people over the age of 65 and accounting for 60%–70% of dementia cases (Burns and Iliffe, 2009). Typically, the AD-prodromal stage presents as mild cognitive impairment (MCI; Stephan et al., 2012) clinically defined as cognitive difficulties beyond those expected based on age and education, but insufficient to interfere with daily activities (Petersen et al., 1999; Petersen, 2004). MCI can present with a variety of symptoms but is termed amnestic MCI (aMCI) in cases where memory loss is the predominant symptom.

In AD, the first neurofibrillary tangles appear in the parahippocampal regions (Stage I) followed later, and accompanied by cognitive symptoms, in the hippocampus formation (stage III; Braak and Braak, 1991a,b, 1995). Understandably, this knowledge has reinforced focus on the hippocampus in the context of memory loss in AD but much less well-known and less well-understood are the appearance of tangles and plaques in the thalamic nuclei in parallel with those in the hippocampus. Their appearance is often characterized as an event downstream of the hippocampus pathology transmitted by the projections of the mammillary bodies, but this view is challenged by metabolic studies indicating that the earliest consistent declines occur not in hippocampus but in posterior cingulate cortex (PCC; Minoshima et al., 1994, 1997) where amyloid deposition is highest (Buckner et al., 2005; Mintun et al., 2006). The thalamus, with its dense network of reciprocal interconnections with both hippocampus and PCC, is therefore implicated by association (Vann et al., 2009; Aggleton et al., 2010).

Such a view is supported by detection of thalamic atrophy in pre-symptomatic familial AD on average 5.6 years prior to expected symptom onset (Ryan et al., 2013) together with increased amyloid burden (Knight et al., 2011a,b) and substantial evidence suggesting that thalamic atrophy is present in MCI prior to AD (Chételat et al., 2005; Shiino et al., 2006; de Jong et al., 2008; Ferrarini et al., 2008; Cherubini et al., 2010; Roh et al., 2011; Pedro et al., 2012; Zhang et al., 2013). Structural irregularities have a sufficient impact on thalamo-cortical circuits to allow healthy subjects to be differentiated from those with MCI through impaired functional integrity (Cantero et al., 2009). Conversely, carriers of the apolipoprotein ε2 allele i.e., those showing a genetic predisposition against developing AD, demonstrate significantly enhanced functional (Patel et al., 2013) and structural (Chiang et al., 2012) integrity of the thalamus.

Analysis of low frequency BOLD signal oscillations have revealed several resting state networks. Of these, the default mode network (DMN; Raichle et al., 2001; Greicius et al., 2003; Damoiseaux et al., 2006) has consistently been identified as dysfunctional in both MCI and AD in the context of amyloid burden (Hedden et al., 2009; Drzezga et al., 2011; Mormino et al., 2011; Sheline et al., 2011) and genetic risk (Roses, 1996; Sheline et al., 2010; Wang et al., 2012; Chhatwal et al., 2013).

The DMN comprises medial prefrontal cortex (mPFC), middle temporal gyrus (MTG), lateral inferior parietal lobes (IPL), PCC and hippocampus regions. These nodes have been identified as important hubs within the cortex (Buckner et al., 2009) whose persistent background activity and dense, long range interconnectivity may facilitate the early deposition and prion-like transmission of amyloid plaques (Wermke et al., 2008; Raj et al., 2012). DMN topography is therefore recapitulated in the pattern of atrophy, hypometabolism and amyloid deposition within the cortex (Buckner et al., 2005, 2008).

Thalamus appears to play a role modulating distributed cortical networks (Di and Biswal, 2014). It is therefore of note, that direct structural connections between the thalamus and DMN (or thalamo-DMN pathway) components have been described *in vivo* using diffusion tensor imaging (DTI; Fernández-Espejo et al., 2012) and that these are sites of atrophy (Zarei et al., 2010). Crucially, lesions to the thalamus are known to cause DMN dysfunction (Jones et al., 2011). One suggestion is that abnormal task-induced deactivation of DMN response patterns in aMCI are a consequence of impaired thalamo-cortical signaling (Pihlajamäki and Sperling, 2009).

The thalamus sends widespread connections to its ipsilateral cortical hemisphere which are returned via cortico-thalamic feedback connections. Together these form a thalamo-cortico-thalamic feedback loop (Sherman and Guillery, 2006; Sherman, 2007; Zhang et al., 2008, 2010). Such an arrangement is critical for generating the ubiquitous oscillations of the cortex recorded by EEG and fMRI but its contribution (and other subcortical components) to regulating the DMN in health and disease is largely unexplored. On this basis, we chose to investigate the impact of impaired thalamo-cortical microscopic white matter anatomy on interactions in the DMN in aMCI patients.

We performed constrained spherical deconvolution (CSD)-based probabilistic fiber tractography of the thalamo-DMN white matter pathways in a cohort of older adults with aMCI and healthy age-matched controls. We also examined the effective connectivity of the resting state thalamo-DMN interactions using a spatio-temporal formulation of Granger Causality (GC). In contrast to simple statistical correlation (i.e., functional connectivity), effective connectivity is more ambitious and attempts to quantify the causal influence one region exerts over another. Given that thalamo-cortical neural signals appear to coordinate distributed networks (Di and Biswal, 2014), such an approach provides greater scope for clarifying the interactions between thalamus and cortex during the transition between health and disease. We predicted that abnormal DMN causal activity would be linked to structural deficits in the thalamo-DMN pathway.

## Materials and Methods

### Participants

Twenty six HC participants and 20 older participants with aMCI took part in the study. The HCs were community-dwelling older adults recruited from the greater Dublin area (Ireland) via newspaper advertisements. They underwent a health screening questionnaire and a neuropsychological assessment, the Consortium to Establish a Registry for Alzheimer’s Disease (CERAD; Morris et al., 1989), in order to rule out possible cognitive impairment before inclusion in the study. The CERAD battery has been shown to be sensitive to the presence of age related cognitive decline (Welsh et al., 1991, 1992). All of the older participants included in the study scored no more than 1.5 SD below the standardized mean scores for subjects of a similar age and education level on any of the sub-tests. The aMCI participants were recruited from memory clinics in St. James Hospital and St. Patrick’s Hospital in Dublin, Ireland, and were diagnosed by a clinician according to the Peterson criteria (Petersen et al., 1999)—i.e., abnormal memory scores for age and education level with no dementia. Four were single amnestic aMCI, and 16 were multi-domain aMCI (Petersen, 2004). Neuropsychological measures were administered or supervised by an experienced neuropsychologist and included the Mini-Mental State Examination (MMSE; Folstein et al., 1975) and Cambridge cognitive examination (Huppert et al., 1995).

All of the participants were right-handed with no history of head trauma, neurological disease, stroke, transient ischemic attack, heart attack, or psychiatric illness. They completed the Geriatric Depression Scale (GSD; Yesavage et al., 1983), the Eysenck Personality Questionnaire Revised Edition Short Scale (EPQ-R; Eysenck and Eysenck, 1994), and a Cognitive Reserve Questionnaire (Rami et al., 2011) before the MRI scan (Table 1). The groups did not differ in terms of age, gender, education level, or levels of cognitive reserve as assessed by the self-report Cognitive Reserve Questionnaire. The aMCI group had lower MMSE scores, higher GDS scores, and scored lower on the EPQ measure of extraversion than the HC group. The study had full ethical approval from the St. James Hospital and the Adelaide and Meath Hospital, incorporating the National Children’s Hospital Research Ethics Committee and St. Patrick’s University Hospital Research Ethics Committee. All subjects gave written informed consent in accordance with the Declaration of Helsinki.

**Table 1:** Results of independent samples *t*-tests, except for gender which was compared with a Fischer’s exact test.

	HC (*n* = 26)	aMCI (*n* = 20)	p* (*df* = 44)
Gender	15 M, 11 F	10 M, 10 F	1.00
Age	69.30 ± 6.35	69.05 ± 7.55	0.90
Ethnicity	White (Irish)	White (Irish)	-
Education	13.38 ± 3.73	14.32 ± 3.02	0.38
MMSE	28.65 ± 0.85	27.05 ± 2.17	**0.0013**
GDS	0.77 ± 1.07	2.58 ± 2.27	**0.0008**
EPQ E	8.04 ± 2.47	5.53 ± 3.37	**0.0061**
EPQ N	2.69 ± 2.43	3.78 ± 3.39	0.21
CR	16.65 ± 3.62	16.58 ± 4.97	0.95

*Standard deviations are indicated. Statistically significant differences are indicated in bold. MMSE, Mini-Mental State Exam; GDS, geriatric depression scale; EPQ E, Eysenck personality questionnaire extraversion scale; EPQ N, Eysenck personality questionnaire neuroticism scale; CR, cognitive reserve scale*.

### MRI Data Acquisition

Whole-brain high angular resolution diffusion imaging (HARDI) data were acquired on a 3.0 Tesla Philips Intera MR system (Best, Netherlands) equipped with an eight channel head coil. A parallel sensitivity encoding (SENSE) approach (Pruessmann et al., 1999) with a reduction factor of two was used during the diffusion weighted image (DWI) acquisition. Single-shot spin echo-planar imaging was used to acquire the DWI data with the following parameters: echo time (TE) = 79 ms, repetition time (TR) = = 20,000 ms, field of view (FOV) = 248 mm, matrix = 112 × 112, isotropic voxel of 2.3 mm × 2.3 mm × 2.3 mm, and 65 slices with 2.3 mm thickness with no gap between the slices. Diffusion gradients were applied in 61 isotropically distributed orientations with *b* = 3000 s/mm^2^, and four images with *b* = 0 s/mm^2^ were also acquired. A high-resolution 3D T1-weighted anatomical image was acquired for each participant with the following parameters: TE = 3.9 ms, TR = 8.5 ms, FOV = 230 mm, slice thickness = 0.9 mm, voxel size = 0.9 mm × 0.9 mm × 0.9 mm. Resting-state fMRI data were also acquired during the scanning session. The scan lasted for 7 min during which time the participants were asked to keep their eyes open and fixate on a cross hairs in the center of a screen behind the MR scanner, visible via a mirror. The BOLD signal changes were measured using a T2*-weighted echo-planar imaging sequence with TE = 30 ms and TR = 2000 ms. Each volume of data covered the entire brain with 39 slices, and the slices were acquired in interleaved sequence from inferior to superior direction. Two-hundred and ten volumes of data were acquired, with voxel dimensions of 3.5 mm × 3.5 mm × 3.85 mm and a 0.35 mm gap between the slices.

### Face-Name Encoding and Recognition Task Protocol

Relationships between the participants’ structural/effective connectivity measures and memory were subsequently examined using data obtained from a face–name recognition task following the resting-state scan. The participants viewed a series of 27 emotional faces (Erwin et al., 1992) with a name presented underneath each one. This task was an implicit memory task, in that the participants later completed a surprise memory tasks to test their retention of both the faces and the face–name pairs, however, at the time of encoding, they were not explicitly asked to remember the face–name pairs. Rather, the participants were instructed to judge whether the names matched or suited the faces. It was explained that this was a subjective decision, with no right or wrong answer. The participants responded yes or no by pressing a button on a MR-compatible response pad held in their right or left hand, respectively, using the index finger of either hand. Each face–name combination was presented for 4 s and was shown twice during the run. The faces were positive, negative, or neutral in valence and there were equal numbers of valence types as well as gender. The presentation of the face–name pairs was grouped according to the emotional valence of the faces. In each instance, a group of either two, three, or four faces of one valence type was presented randomly using an event-related paradigm, subsequently, there was a delay during which a white cross hair was presented (control condition). The duration of the white cross was varied according to the duration of the face stimulus. For instance, if a single face was presented for 4 s the subsequent white cross was also shown for 4 s and then the next block of faces began. The stimuli were delivered using Presentation v.16.1 (Neurobehavioral Systems, Albany, CA, USA).

Approximately 15 min following the encoding phase, the participants performed a short computer-based recognition task. The emotional faces were presented one at a time on a black background with three names underneath. One of the names was the correct name; one name was a name that had been paired with a different face (distractor; incorrect name), while the third name was a new name (foil; incorrect name). The participants responded by pressing a button on the left, middle, or right side of a keyboard to correspond with the relative position of the name on the screen. The stimuli were presented for 5 s and followed by an inter-trial interval of 5 s. This longer trial length was to facilitate performance of this task as it was quite challenging. Before the task began, the participants completed a short practice run of five trials.

### Resting State Pre-Processing

FMRI data processing was carried out using FMRI Expert Analysis Tool (FEAT) Version 6.00, part of FMRIB’s Software Library (FSL)^1^. Registration to high resolution structural and/or standard space images was carried out using FNIRT (Andersson et al., 2007). The following pre-statistics processing was applied; motion correction using MCFLIRT (Jenkinson et al., 2002), slice-timing correction using Fourier-space time-series phase-shifting, non-brain removal using BET (Smith, 2002), spatial smoothing using a Gaussian kernel of FWHM 3.0 mm, grand-mean intensity normalization of the entire 4D dataset by a single multiplicative factor, highpass temporal filtering (Gaussian-weighted least-squares straight line fitting, with sigma = 50.0 s).

### Resting State Effective Connectivity

GC is a standard statistical tool for detecting the directional influence one system component exerts over another. The concept, originally introduced by Wiener (1956), and later incorporated into a data analysis framework by Granger (1969) is described as follows. If historical information from time series X significantly improves prediction accuracy of the future of time series Y in a multivariate autoregressive model (MVAR), GC is identified. This may be viewed as a measure of model prediction error where GC quantifies the reduction in prediction error when past values of X are included in the explanatory variables of Y (Schelter et al., 2006). By fitting a time invariant MVAR model to the experimental time series the classic GC formulation ignores crucial time-varying properties of the system. Such an approach makes the tacit assumption that the longer the time series, the more reliable the GC estimates. While this may be correct in static circuit representations (Smith et al., 2011), under time-varying conditions this principle is no longer valid. A more robust method is to divide the time series into equal windows and consider them separately. Here, an optimal trade-off between the length of the time windows and the accuracy of the estimated coefficients for each window must be determined. Time windows that are too short prevent the accurate estimation of parameters, while time windows that are too long increase the probability of incorrect inferences of GC. Accordingly, the current article utilizes a novel spatio-temporal GC formulation to quantify the effective connectivity changes between region of interest (ROI; Luo et al., 2013). In this framework, finding the optimal time window length reduces to the solution of a constrained optimization problem,

$$min_{l_{0}}{}_{\left(m\right)}\left(GC_{\text{err}}(l_{0}(m))+\frac{1}{GC_{avg}(l_{0}(m))}\right)$$

where we seek to simultaneously minimize model prediction error *GC*_err_ (i.e., the weighted average of the variances of the residuals in each time window) and maximize detected causality information *GC*_avg_ (i.e., the average GC over all time windows). This is performed for time windows of different length *l*_o_(*m*) = *t*_1_, …, *t_m_*. The time window producing the lowest Bayesian information criterion (BIC) is considered optimal. By considering optimal time windows, the spatio-temporal framework allows a more reliable and precise estimate of GC in experimental datasets with time varying properties. This approach has been shown to yield more accurate estimates of GC on resting state fMRI data than traditional GC metrics. In this case, the last 208 time points for each region under consideration were extracted from the functional image volume and divided into four windows with the first two time points removed to avoid start up transients. In terms of spatial resolution, GC is calculated between all pairs of voxels from the two ROI under consideration. The mean GC among all pairs of voxels was then used as the final estimate.

### CSD White Matter Tractography Using MRtrix3

A method for controlling free water contamination of tissue and the resultant partial volume effects is especially important around the fornix where atrophy and cerebrospinal fluid (CSF) is prevalent. The free water elimination technique (Pasternak et al., 2009) has been successfully applied in previous tractography studies of ageing and aMCI (Metzler-Baddeley et al., 2012a,b; Fletcher et al., 2014; Kehoe et al., 2015) however at higher b-values the Gaussian assumption underlying the bi-tensor model is no longer valid and a more simple heuristic is indicated. Accordingly, we use the standard free water elimination approach to identify and mask voxels with high free water content but fit the conventional DTI model to each voxel.

The *dwipreproc* preprocessing script was to perform eddy current-induced distortion and motion correction using the FSL tool eddy (Andersson and Sotiropoulos, 2016). The standard MRtrix3 processing script *dwibiascorrect* was used to eliminate low frequency intensity inhomogeneities across the DWI series. The script uses bias field correction algorithms available in the FSL software package (Zhang et al., 2001).

Probabilistic white matter tractography was performed on the DWIs using the MRtrix3 software package^2^. Crossing fibers were resolved using the CSD algorithm (Tournier et al., 2004, 2012). MRtrix3 pre-processing included computing the diffusion tensors images (or diffusion ellipsoids) for each voxel from which the fractional anisotropy (FA), axial (DA), radial (RD) and mean (MD) diffusivity.

Whole-brain tractography was performed using every voxel as a seed point. The principle diffusion orientation at each point was estimated by the CSD tractography algorithm, which propagated in 0.1 mm steps along this direction. At each new location the fiber orientation(s) was estimated before the tracking moved a further 0.1 mm along the direction that subtended the smallest angle to the current trajectory. A trajectory was followed through the data until the scaled height of the fiber orientation density function peak dropped below the default threshold, or the direction of the pathway changed through an angle of more than 90°.

**Figure 1:**
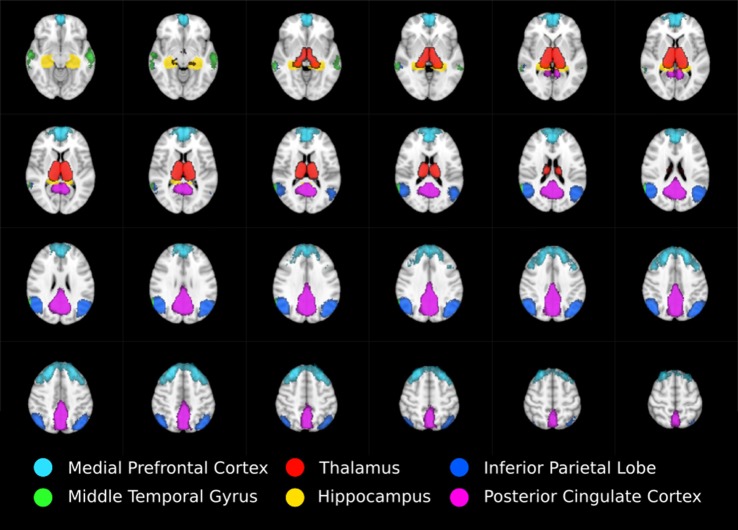
Regions of interest moving from inferior (top left) to superior (bottom right) defining thalamo-DMN white matter tractography masks. DMN components mPFC, MTG, IPL, and PCC were defined using probabilistic template (Wang et al., 2014) while thalamus and hippocampus were defined using the Harvard-Oxford subcortical structural atlas.

Anatomical masks were used to divide the results into circumscribed regions. The DMN was defined by probabilistic template (Wang et al., 2014) and the hippocampus and thalamus using the Harvard-Oxford subcortical structural atlas (Figure 1). Streamlines beginning in one mask and terminating in another were considered in a pairwise fashion for all ROI. In addition, the FSL tool FMRIB’s Automated Segmentation Tool (FAST) was used to derive a white matter brain mask to constrain tractography. Any tracks tracts exiting the white matter were considered spurious and discarded. Tracts were prevented from propagating between hemispheres by a stop region placed down the midline corresponding to the corpus callosum.

Statistically significant differences in the mean FA, DA, RD and MD of tracks in HC vs. aMCI were tested by way of a two tailed two sample *t*-test at *p* < 0.05 corrected for multiple comparisons.

### Independent Component Analysis (ICA)

The DMN was identified for each subject using ICA. Analysis was carried out using Probabilistic ICA (Beckmann and Smith, 2004) as implemented in Multivariate Exploratory Linear Decomposition into Independent Components (MELODIC) Version 3.14, part of FSL. The following data pre-processing was applied to the input data: masking of non-brain voxels, voxel-wise de-meaning of the data, normalization of the voxel-wise variance. Pre-processed data were whitened and projected into a 62-dimensional subspace using probabilistic Principal Component Analysis where the number of dimensions was estimated using the Laplace approximation to the Bayesian evidence of the model order (Minka, 2001; Beckmann and Smith, 2004). The whitened observations were decomposed into sets of vectors which describe signal variation across the temporal domain (time-courses) and across the spatial domain (maps) by optimizing for non-Gaussian spatial source distributions using a fixed-point iteration technique (Hyvärinen, 1999). Estimated component maps were divided by the standard deviation of the residual noise and thresholded by fitting a mixture model to the histogram of intensity values (Beckmann and Smith, 2004). The number of components was automatically estimated. The component corresponding to the DMN was selected by cross correlating all the components with a probabilistic DMN template (Wang et al., 2014). The fMRI BOLD signal was extracted from DMN components mPFC, MTG, IPL and PCC, combined with those extracted from hippocampus and thalamus masks, and analyzed using the spatio-temporal GC method to determine the effective connectivity. A standard two tailed *t*-test was used to determine significant differences between the HC and aMCI patients at *p* < 0.05 corrected for multiple comparisons.

## Results

### Comparison of Resting State Thalamo-DMN Effective Connectivity in HC vs. aMCI Subjects

The spatio-temporal GC effective connectivity analysis revealed significant differences in a circumscribed set of regions at the Bonferroni corrected threshold of *p* < 0.0014.

**Figure 2:**
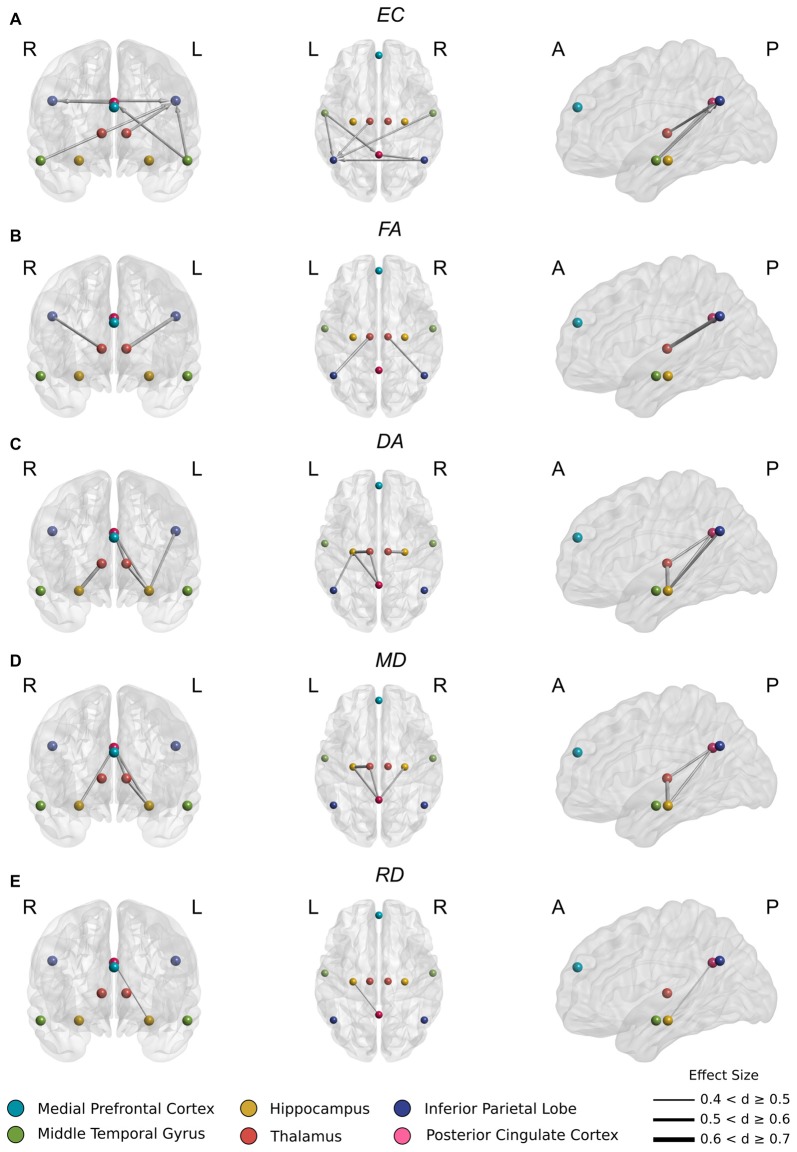
**(A)** Significantly reduced incoming effective connectivity to left IPL from thalamus and posterior DMN nodes. **(B)** Significantly reduced FA in thalamo-IPL tracts where the magnitude of reduction corresponded to the degree of effective connectivity disruption in **(A)**. **(C)** Significantly reduced DA in the left Papez circuit including hippocampo-thalamus, thalamo-PCC and PCC-hippocampal tracts. **(D)** As in **(C)**, significantly reduced MD in the left Papez circuit. **(E)** Significantly reduced RD in left hippocampo-PCC tracts.

In aMCI, several incoming connections to PCC and left IPL showed reduced casual connectivity. An especially pronounced decrease in causal interaction to left IPL from other DMN components, hippocampus, and thalamus was observed (Figure 2A). Reduced connectivity to left IPL included incoming connections from left thalamus (*t*_(44)_ = 3.77, *p* < 0.001), left (*t*_(44)_ = 4.3, *p* < 0.0001) and right (*t*_(44)_ = 3.80, *p* < 0.001) MTG and from right IPL (*t*_(44)_ = 3.83 *p* < 0.001). These changes correspond to a highly significant (*t*_(44)_ = 5.10, *p* < 0.00001) decrease in average FA in the white matter between left thalamus and left IPL (Figure 2B).

Also in aMCI, significant reductions in connectivity to PCC from left MTG (*t*_(44)_ = 3.93, *p* < 0.001) were found, together with significant reductions in connectivity to right IPL from PCC (*t*_(44)_ = 3.73, *p* < 0.001) (Figure 2A).

### Comparison of Thalamo-DMN Microstructural Integrity in HC vs. aMCI Subjects

**Figure 3:**
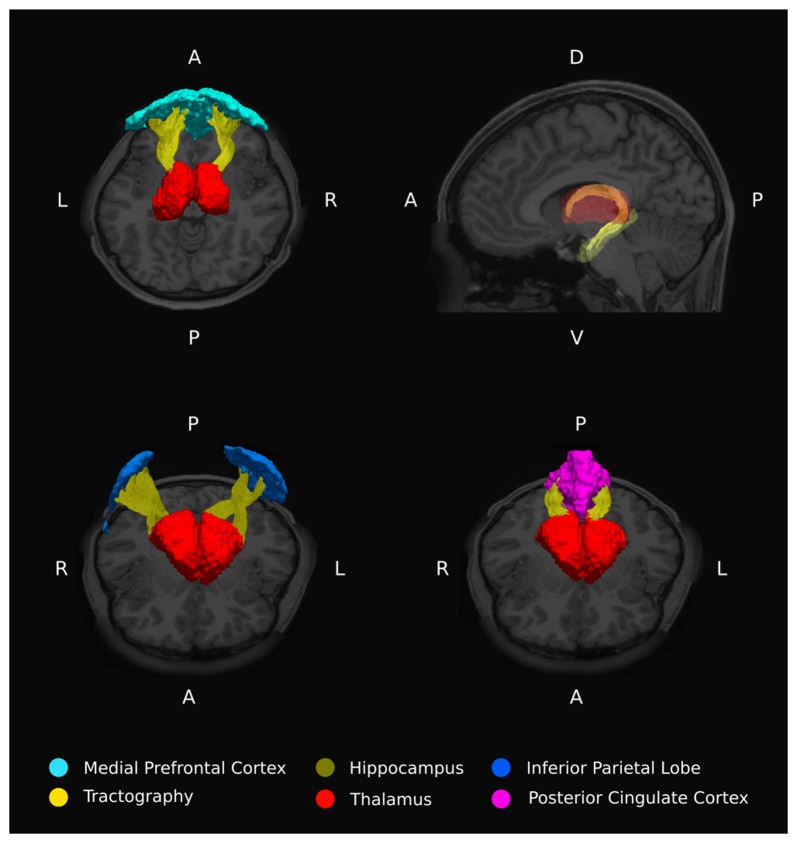
Example thalamo-DMN white matter tracts from a single representative healthy subject.

In aMCI, CSD white matter tractography identified statistically significant increases at the Bonferroni corrected threshold in average DA, RD and MD in the white matter fiber pathways connecting thalamus to hippocampus, PCC and IPL (Figure 3). Significant decreases in average FA were also detected in the white matter between thalamus and IPL. These included:

Significant decreases in FA (Figure 2B) between right thalamus and right IPL (*t*_(44)_ = 3.81, *p* < 0.001) and between left thalamus and left IPL (*t*_(44)_ = 5.24, *p* < 0.00001).

Significant increases in DA (Figure 2C) between right thalamus and right hippocampus (*t*_(44)_ = −4.68, *p* < 0.0001), right hippocampus and PCC (*t*_(44)_ = −4.02, *p* < 0.001), left thalamus and left hippocampus (*t*_(44)_ = −5.33, *p* < 0.00001), left thalamus and PCC (*t*_(44)_ = −3.91, *p* < 0.001), left hippocampus and left IPL (*t*_(44)_ = −4.13 *p* < 0.001), and left hippocampus and PCC (*t*_(44)_ = −3.91, *p* < 0.001).

These changes were recapitulated in the MD metric (Figure 2D) with significant increases between right hippocampus and PCC (*t*_(44)_ = −3.69, *p* < 0.001), left thalamus and left hippocampus (*t*_(44)_ = −4.34, *p* < 0.0001), left thalamus and PCC (*t*_(44)_ < −3.63, *p* = 0.001) and left hippocampus and PCC (*t*_(44)_ = −4.17, *p* < 0.001).

Finally, a significant increase in RD (Figure 2E) between left hippocampus and PCC (*t*_(44)_ = −3.66, *p* < 0.001) was also found.

### Empirical Measures of Effective and Structural Connectivity Predict Memory Performance

To investigate whether empirical measures of effective and structural connectivity relate to memory, we regressed the diffusivity and GC metrics against the results from a face-name encoding and recognition task using gender, age and motion parameter estimates as covariates of no interest.

**Figure 4:**
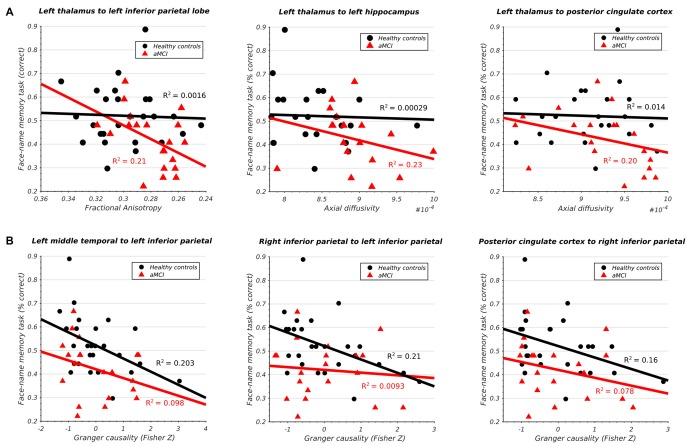
**(A)** Significant association between structural integrity of thalamo-cortical white matter pathways and memory performance in aMCI subjects. **(B)** Significant association between inferior parietal lobe effective connectivity and memory performance in HCs.

The aMCI cohort displayed a significant negative correlation between the integrity of the left thalamo-cortical white matter connectivity and memory in three DMN regions (Figure 4A) including IPL (*t*_(24)_ = −2.43, *p* < 0.05), hippocampus (*t*_(24)_ = −2.31, *p* < 0.05), and PCC (*t*_(24)_ = −2.21, *p* < 0.05). The healthy subjects displayed no such relationship.

Conversely, the healthy cohort demonstrated a significant negative correlation between the effective connectivity of IPL and memory and three other DMN regions (Figure 4B) including left MTG (*t*_(24)_ = −2.47, *p* < 0.05), right IPL (*t*_(24)_ = −2.54, *p* < 0.05), and PCC (*t*_(24)_ = −2.21, *p* < 0.05). The same relationship was absent in the aMCI cohort. All results survived multiple-comparison correction with FDR (*q* < 0.1).

## Discussion

The appearance of atrophy, tangles and plaques in thalamus is often characterized as a secondary process resulting from atrophy in the hippocampus and the prion-like transmission of pathology along the white matter topography (Raj et al., 2012). But such a view is inconsistent with evidence suggesting that the earliest metabolic changes occur not in hippocampus but in posterior DMN node PCC. Thus, structural deficits in thalamus may be driving early PCC hypometabolism and initiating the cascade of DMN functional anomalies typically associated with early AD. Accordingly, we used a multimodal approach to assess the impact of thalamo-cortico-thalamic feedback loop integrity on DMN functionality in aMCI.

We found significant structural abnormalities in the thalamo-PCC and thalamo-IPL white matter fiber pathways in the aMCI cohort (Figures 2B–E). A pronounced reduction in left thalamo-IPL effective connectivity (Figure 2A) corresponded with significant thalamo-IPL structural impairment (Figure 2B). Critically, the integrity of thalamic white matter and memory was correlated in the aMCI cohort but not in the HCs (Figure 4A). In general, the gradient of structural impairment followed a hippocampo-thalamo-PCC axis consistent with a prion-like dissemination of pathology (Raj et al., 2012) along the major white matter fiber pathways of the Papez circuit (Papez, 1937). No structural abnormalities were identified between cortical DMN components mPFC, MTG, IPL and PCC, however significant disruption to incoming IPL effective connectivity was observed (Figure 2A), and this distinguished HC and aMCI memory performance (Figure 4B).

Overall, our findings are broadly suggestive. One interpretation is that disrupted effective connectivity in posterior DMN nodes PCC and IPL is, to some extent, inspired by incipient thalamo-cortical deafferentation. If true, this finding may help explain abnormal task-induced DMN response patterns typically found in aMCI and AD subjects (Pihlajamäki and Sperling, 2009).

### Impaired Hippocampo-Thalamo-PCC White Matter Anatomy and Abnormal PCC Effective Connectivity

The current article identified significant structural impairment between fiber pathways connecting hippocampus and thalamus (Figures 2C,D), thalamus and PCC (Figures 2C,D), and PCC and hippocampus (Figures 2C–E). Measures of left thalamo-cortical structural integrity (including tracts to hippocampus and PCC) correlated with memory performance in the aMCI cohort but not in the HCs (Figure 4A).

Impaired structural relations within the hippocampo-thalamo-PCC complex are likely mediated by their close anatomical association. Together these structures comprise a limbic-diencephalic memory network (Nestor et al., 2003) connected through the circuit of Papez (1937). This structure runs from hippocampus through fornix to anterior thalamus via mammillary bodies and onto PCC before returning to hippocampus to complete the circuit. Interestingly, the current study identified a decreasing gradient of structural impairment between hippocampus, thalamus, and PCC, suggesting that structural deafferentation of PCC through impaired hippocampus and thalamus fiber pathways, likely stems from pathology and atrophy originating in the hippocampal complex. Such a view is consistent with postmortem studies indicating that thalamic nuclei connected to hippocampus are a site of primary degeneration in AD (Xuereb et al., 1991).

Previous work has highlighted a staged disconnection process occurring both along the cingulum bundle between hippocampus and PCC (i.e., the direct route) and within the memory circuit of Papez encompassing thalamic intermediaries (i.e., the indirect route; Villain et al., 2008). Such findings are consistent with early PCC hypometabolism (Matsuda, 2001; Valla et al., 2001; Mosconi et al., 2008; Zhu et al., 2013; Mutlu et al., 2016) where it frequently presents before clinical diagnosis (Minoshima et al., 1997; Johnson et al., 1998) as part of a constellation of metabolic effects focused around medial temporal lobe and thalamus, when memory loss is still a relatively isolated feature (Nestor et al., 2003). Interestingly, PCC hypometabolism appears to correlate with remote hippocampus atrophy early in MCI but transition to both remote and local effects over the course of progression to AD (Teipel and Grothe, 2016).

The current study identified a significant correlation between the structural integrity of hippocampo-thalamus and thalamo-PCC fiber pathways (i.e., the indirect route) and memory in the aMCI cohort which was absent in the HCs (Figure 4A). A similar pattern was identified in the hippocampo-PCC fiber pathway (i.e., the direct route) however this did not survive correction for multiple comparisons. Dysfunction of structures along the hippocampal output pathways to PCC have been linked to episodic memory impairment (Yakushev et al., 2011).

The PCC’s hub status (Hagmann et al., 2008) may predispose to amyloid deposition, atrophy, and hypometabolism (Buckner et al., 2005, 2009) where remote often diffuse damage accumulates as altered PCC connectivity through a form of diaschisis (Meguro et al., 1999; Leech and Sharp, 2014). One suggestion is that direct thalamo-PCC (Figures 2C,D) and distal thalamo-IPL (Figure 2B) white matter structural deficits operate in tandem to initiate a cascade of aberrant effective connectivity in PCC (Figure 2A). Taken together, these findings are consistent with a progressive disconnection of PCC from downstream cortical and subcortical sources with differential effects operating on the direct vs. indirect hippocampo-PCC pathways.

### Impaired Thalamo-IPL White Matter Anatomy and Abnormal IPL Effective Connectivity

The current article identified significant impairments in thalamic white matter circuitry serving bilateral IPL where the magnitude of diffusivity change (Figure 2B) correlated with the intensity and extent of effective connectivity disruption in each hemisphere (Figure 2A).

Marked structural deficits were observed in left thalamo-IPL white matter connectivity together with significantly reduced effective connectivity from left thalamus. In the aMCI cohort, measures of reduced thalamo-IPL structural integrity correlated with memory performance (Figure 4A). Left thalamo-IPL structural abnormalities were accompanied by widespread decreases in effective connectivity from other DMN regions. Similarly, in right hemisphere, thalamo-IPL structural deficits coocurred with disrupted incoming and outgoing IPL effective connectivity. Crucially, the relationship between IPL effective connectivity and memory was disrupted in the aMCI subjects but not in the HCs (Figure 4B).

Several converging findings implicate the pulvinar nucleus of the thalamus in this dysfunction. The pulvinar nuclei appear to play a role in cortico-cortical communication where they receive driving input from IPL and relay signals back to cortex via ascending thalamo-cortical projections (Saalmann et al., 2012). Since direct cortico-cortical projections far outnumber projections to pulvinar nucleus from cortex, the pulvinar is unlikely to be the primary route for the transfer of cortico-cortical sensory signals, rather, it may act to coordinate interactions between distributed cortical networks as a function of attention (Basso et al., 2005). Interestingly, entorhinal cortex connects directly to pulvinar nucleus via a non-fornical temporopulvinar tract (Saunders et al., 2005; Zarei et al., 2013) which may provide a conduit for the prion-like transsynaptic spread of disease agents originating in hippocampus (Raj et al., 2012). Consistent with this hypothesis, the present study identified significant structural impairment between thalamus and hippocampus (Figures 2C,D).

Taken together, these findings are consistent with the idea that disrupted posterior DMN node effective connectivity is, to some extent, mediated by impaired thalamo-cortical white matter circuitry.

### Methodological Considerations

Some limitations should be noted. The major weakness of the article is that each thalamic nucleus has specific cortical connections and functions, yet the present analysis uses a holo-thalamic approach. It would be more informative to determine whether sub-nuclei show differential causal interactions between specific regions of thalamus and crucial DMN nodes and likewise, whether these connections show varying degrees of structural impairment. Such an approach would reveal the specificity of AD pathology for individual thalamic nuclei. Analyzing the entire thalamus may dilute these results. Our findings should therefore be considered as preliminary evidence warranting further investigation.

It should also be noted that the indirect relationship between fMRI BOLD signal and the underlying neural mechanism is especially problematic when applying GC and should be noted as a weakness in the present study. First, the study’s sampling rate (repetition time or TR) of 2 s is considerably slower than the millisecond temporal resolution of the neuronal activity we seek to qualify. Second, the temporal precedence assumptions of GC can be violated by regional differences in the latency of the hemodynamic response (Handwerker et al., 2004; Friston, 2011). Since neurovascular coupling can be altered in complex ways by disease, the likelihood of such an event is magnified in the aMCI patient cohort (Handwerker et al., 2012). One typical scenario, is that region X causally influences Y at the neuronal level but has a longer time-to-peak in its HRF due to pathology of the neurovasculature. Thus, GC analysis of BOLD signal may incorrectly suggest that Y is causally implicated in causing X. Simulations show that GC performs well when the HRF delay between regions is short (Deshpande et al., 2010; Schippers et al., 2011) however sufficiently fast sampling, on par with the neuronal delays themselves, is required to ensure GC is fully invariant to HRF latency (Seth et al., 2013). Other simulations suggest that the relationship between GC at the neuronal level and GC at the fMRI level is reasonably preserved over a range of sampling rates and convolution parameters (Wen et al., 2013). Whatever the case, sub-second temporal resolutions have been made available (Feinberg et al., 2010; Feinberg and Yacoub, 2012) and are standard as part of the Human Connectome Project (Van Essen et al., 2013). The most recent advances enable a temporal resolution as fast as 50 ms (Boyacioğlu and Barth, 2013).

Critically, GC makes no claims regarding the underlying physical mechanisms responsible for the observed differences in causal relationships between regions. In contrast, the dynamic causal modeling approach (DCM; Di and Biswal, 2014) explicitly specifies dynamic effective relationships at the neuronal level, allowing the most likely structural model for generating the observed data to be identified. Applying DCM in future studies will help clarify thalamic involvement in posterior DMN dysfunction.

The choice of CSD-based tractography reflects the growing recognition that assumptions underlying the DTI model may not always be met in practice (Wheeler-Kingshott and Cercignani, 2009; Jones, 2010; Jones and Cercignani, 2010). The DTI model can only capture a unitary fiber direction within a single voxel despite observations that 90% of the brain is composed of multiple crossing fibers (Jeurissen et al., 2013). For this reason CSD attempts to map several fiber directions per voxel by taking advantage of the high number of diffusion encoding directions and large b-values acquired using the HARDI acquisition protocol (Tournier et al., 2007, 2008; Mielke et al., 2012; Farquharson et al., 2013). Using large b-values has an additional advantage. By allowing a sufficiently long diffusion path to be measured water molecules are more likely to collide with their container. This may be relevant in patients with neurodegenerative disorders who have increased permeability of membranes, greater extracellular space due to axonal atrophy, demyelination and glial pathology (Acosta-Cabronero and Nestor, 2014). To date, only a handful of tractography studies have utilized HARDI data and large b-values (Thiebaut de Schotten et al., 2011; Meng et al., 2013; Yeatman et al., 2014; Xie et al., 2015) and only one specifically in clinical aMCI and AD (Kehoe et al., 2015).

The absence of indirect biomarkers of AD pathology (CSF biomarkers and/or amyloid PET imaging) should also be acknowledged as a weakness in the present article.

## Conclusion

The dynamic nature of thalamo-cortical dialog suggests that abnormalities in DMN operation may best be understood from the perspective of thalamic dysfunction. The present study employed diffusion imaging and effective connectivity to clarify the relationship between the physical integrity of thalamic white matter projections and the activity of the DMN. Significant changes in the diffusivity metrics of thalamic white matter projection tracts to hippocampus, PCC and IPL (Figures 2B–D) were identified. Effective connectivity changes corresponding to the same regions were also observed (Figure 2A). Interestingly, no structural deficits were found between DMN nodes suggesting that early changes in DMN activity could be a result of impaired thalamo-cortical structural integrity.

Such a conclusion is supported by previous resting state MEG (Garcés et al., 2014), EEG (Schreckenberger et al., 2004; Garcés et al., 2013; Moretti, 2015) and fMRI (Greicius et al., 2004; Sorg et al., 2007; Damoiseaux et al., 2012) studies citing disruption in posterior thalamo-cortical alpha sources. Significant evidence suggests that thalamo-cortical circuitry underlie the generation and modulation of alpha and theta rhythms and that average power is attenuated in these frequency bands for MCI and AD subjects (Jeong, 2004; Koenig et al., 2005; Jelles et al., 2008; Park et al., 2008). Several recent modeling studies have proposed a candidate mechanism citing impairment to thalamic reticular fibers in MCI and AD as the source of the dysfunction (Bhattacharya et al., 2011, 2013; Li et al., 2011; Abuhassan et al., 2014).

A corollary of this discussion is the extent to which cortical activity is dependent on cortico-cortical verses thalamo-cortical connections. It has been suggested that thalamic nuclei coordinate distributed cortical regions through cortico-thalamo-cortical pathways. Abnormalities originating in thalamic to PCC and IPL white matter may therefore be sufficient to engender posterior DMN dysfunction without appealing to comparable deficits in cortico-cortical tracts between DMN nodes. Such a view is consistent with the anatomy and timeline of pathogenesis with thalamic nuclei demonstrating pathology an earlier stage of the disease than cortex. Cortical atrophy may therefore be in response to thalamic white matter disruption with commensurate causal abnormalities occurring in response to changes in thalamo-cortical signaling rather than being instigated by structural changes within the cortex. Importantly, the present study is unable to confirm this hypothesis. Other scenarios, in which cortical pathology is causing a degeneration of thalamo-cortical tracts is also possible or likewise, a parallel disruption in both thalamus and cortex.

Overall, these results provide a compelling and previously unexplored physical basis for posterior DMN dysfunction and abnormal fMRI task-induced deactivation response patterns in aMCI and AD patients and underscore the need to consider neurodegenerative changes within a wider system context including contributions of both cortical and subcortical thalamic components. This work complements a growing body of evidence that suggests effective connectivity is disrupted in neurodegenerative disorders such as aMCI and AD and that these changes are underpinned by structural deficits. For these reasons, joint effective and structural studies will play an increasingly important role in the future as we seek to understand how pathological changes in structural connectivity are reflected in altered network effective connectivity.

## Author Contributions

TA: analysis, manuscript preparation. DC, ALWB and LM: supervisory support. EK, DF, BL, RAK and DL: data collection.

## Conflict of Interest Statement

The authors declare that the research was conducted in the absence of any commercial or financial relationships that could be construed as a potential conflict of interest.

## Footnotes

^1^www.fmrib.ox.ac.uk/fsl

^2^http://www.mrtrix.org/
